# The Association of C-Reactive Protein and *CRP* Genotype with Coronary Heart Disease: Findings from Five Studies with 4,610 Cases amongst 18,637 Participants

**DOI:** 10.1371/journal.pone.0003011

**Published:** 2008-08-20

**Authors:** Debbie A. Lawlor, Roger M. Harbord, Nic J. Timpson, Gordon D. O. Lowe, Ann Rumley, Tom R. Gaunt, Ian Baker, John W. G. Yarnell, Mika Kivimäki, Meena Kumari, Paul E. Norman, Konrad Jamrozik, Graeme J. Hankey, Osvaldo P. Almeida, Leon Flicker, Nicole Warrington, Michael G. Marmot, Yoav Ben-Shlomo, Lyle J. Palmer, Ian N. M. Day, Shah Ebrahim, George Davey Smith

**Affiliations:** 1 MRC Centre for Causal Analyses in Translational Epidemiology, University of Bristol, Bristol, United Kingdom; 2 Department of Social Medicine, University of Bristol, Bristol, United Kingdom; 3 Division of Cardiovascular and Medical Sciences, University of Glasgow, Glasgow, United Kingdom; 4 Department of Epidemiology and Public Health, The Queen's University of Belfast, Belfast, United Kingdom; 5 Department of Epidemiology and Public Health, University College London, London, United Kingdom; 6 School of Surgery and Pathology, University of Western Australia, Perth, Australia; 7 School of Population Health, University of Queensland, Herston, Queensland, Australia; 8 School of Medicine and Pharmacology, University of Western Australia, Perth, Australia; 9 Western Australian Centre for Health & Ageing, University of Australia, Perth, Australia; 10 Laboratory for Genetic Epidemiology, Western Australian Institute for Medical Research and University of Western Australia Centre for Medical Research, University of Western Australia, Perth, Australia; 11 Department of Epidemiology & Population Health, London School of Hygiene & Tropical Medicine, London, United Kingdom; University of Newcastle, United Kingdom

## Abstract

**Background:**

It is unclear whether C-reactive protein (CRP) is causally related to coronary heart disease (CHD). Genetic variants that are known to be associated with CRP levels can be used to provide causal inference of the effect of CRP on CHD. Our objective was to examine the association between *CRP* genetic variant +1444C>T (rs1130864) and CHD risk in the largest study to date of this association.

**Methods and Results:**

We estimated the association of *CRP* genetic variant +1444C>T (rs1130864) with CRP levels and with CHD in five studies and then pooled these analyses (N = 18,637 participants amongst whom there were 4,610 cases). CRP was associated with potential confounding factors (socioeconomic position, physical activity, smoking and body mass) whereas genotype (rs1130864) was not associated with these confounders. The pooled odds ratio of CHD per doubling of circulating CRP level after adjustment for age and sex was 1.13 (95%CI: 1.06, 1.21), and after further adjustment for confounding factors it was 1.07 (95%CI: 1.02, 1.13). Genotype (rs1130864) was associated with circulating CRP; the pooled ratio of geometric means of CRP level among individuals with the TT genotype compared to those with the CT/CC genotype was 1.21 (95%CI: 1.15, 1.28) and the pooled ratio of geometric means of CRP level per additional T allele was 1.14 (95%CI: 1.11, 1.18), with no strong evidence in either analyses of between study heterogeneity (I^2^ = 0%, p>0.9 for both analyses). There was no association of genotype (rs1130864) with CHD: pooled odds ratio 1.01 (95%CI: 0.88, 1.16) comparing individuals with TT genotype to those with CT/CC genotype and 0.96 (95%CI: 0.90, 1.03) per additional T allele (I^2^<7.5%, p>0.6 for both meta-analyses). An instrumental variables analysis (in which the proportion of CRP levels explained by rs1130864 was related to CHD) suggested that circulating CRP was not associated with CHD: the odds ratio for a doubling of CRP level was 1.04 (95%CI: 0.61, 1.80).

**Conclusions:**

We found no association of a genetic variant, which is known to be related to CRP levels, (rs1130864) and having CHD. These findings do not support a causal association between circulating CRP and CHD risk, but very large, extended, genetic association studies would be required to rule this out.

## Introduction

It remains unclear as to whether C-reactive protein (CRP) is causally related to coronary heart disease (CHD). Higher levels of CRP are associated with known risk factors for CHD, and these might confound the purported causal link between CRP and CHD.[1]–[6] Furthermore, it is possible that reverse causality-where either CHD risk factors or pre-symptomatic CHD raise the circulating level of CRP–explains at least some of the association.[7] Whilst confounding and reverse causality might mean that the association seen in observational studies overestimates the true causal association, attenuation by errors (also known as regression dilution bias) might have resulted in an underestimate of the true causal association of CRP with CHD in these studies.

It has been suggested that the exploitation of the principles of Mendelian inheritance can be used to determine unconfounded and unbiased estimates of associations between non-genetic risk factors and disease outcomes,[8]–[10] and that this “Mendelian randomization” approach could provide useful insights into the nature of the association between CRP and CHD.[11] In this approach, the association of a genotype that influences the modifiable risk factor of interest (in this case CRP) with outcome (CHD) is explored. Since heritable units are randomly assigned at conception, genotypes within them should not be associated with confounding factors, such as smoking and socioeconomic circumstances, nor will the genotype be affected by disease processes that influence CRP levels.[8]–[10] Thus, the association between a genotype that is associated with circulating CRP levels and CHD provides a robust test of whether circulating CRP is causally related to CHD. A test that will not be biased by confounding, reverse causality or attenuation by errors (regression dilution bias).[8]–[10]

This approach was used by Casas et al.[12] to assess the association between CRP level and CHD among 3155 European men (985 CHD cases). That study suggested that there was no strong evidence for a causal association between CRP levels and CHD but the authors acknowledged that pooling of larger studies was required to increase confidence in this conclusion.[12] A number of other studies, which have not directly employed Mendelian randomization approaches and have included between 210 to 1062 CHD cases, have also found genetic variants within the *CRP* gene to be unrelated to prevalent and incident CHD events, despite these variants being associated with CRP levels.[13]–[17]

In a recent prospective nested case control study there were no associations between four out of five common haplotypes in *CRP* with CHD risk, despite associations of these haplotypes with CRP levels.[13] The only haplotype that was associated with CHD risk in that study showed an association in the opposite direction to that predicted by its association with CRP levels; the haplotype was associated with lower CRP levels but greater CHD risk.[13] In another study that typed 7 SNPs in *CRP* there were no associations with CHD events except in one sub-group analysis: AA genotype of the triallelic SNP rs3091244 was associated with prevalent coronary heart disease in non-Hispanic white individuals.[18] Such sub-group analyses should be treated with caution unless replicated in independent samples. Finally, Lange and colleagues found differential associations of 4 SNPs in *CRP* with cardiovascular disease events.[19] One SNP (1919A/T) was associated with non-fatal stroke and all cardiovascular disease mortality in white participants, but was not associated with other cardiovascular outcomes (including CHD and carotid intima media thickness) in whites or with any cardiovascular outcomes in Afro-American participants.[19] A second SNP (790A/T) was associated with acute myocardial infarction in Afro-American participants only.[19]

In addition to its importance in understanding the causal role of CRP with CHD, it has also been suggested that determining the association of variation in *CRP* with CHD may be beneficial in its own right.[20] In a recent review, Hage and Szalai noted the paucity and inconsistencies of studies examining SNPs in *CRP* with cardiovascular endpoints, but suggested that if it could be established that variants in *CRP* were robustly associated with CHD events then *CRP* gene profiling could have clinical utility in disease prediction.[20]

The aim of the present study, and a companion paper,[21] is to expand previous work that has attempted to use genetic variation in *CRP* to provide causal inference about the effect of CRP on CHD and to explore whether there is likely to be potential for using *CRP* gene profiling in the prediction of CHD. Ours is the largest analyses to date to examine the association of variation in any *CRP* SNP or haplotype with CHD (we examine association with one SNP rs1130864); our analysis includes 18,637 participants with 4,610 CHD cases. The companion paper uses data from one of the 5 studies included here to examine the association of genetic variation in *CRP* with carotid intima media thickness.[21] Thus, that paper answers a related important question concerned with the nature of the association of CRP with atherogenesis. If our paper shows an association of variation in *CRP* SNP rs1130864 with CHD then the companion paper will contribute to understanding whether this association is due to an association of genotype with atherogenesis, plaque rupture or both.

## Methods

Data from the British Women's Heart and Health Study (BWHHS),[22] the Caerphilly study,[23] the Speedwell study,[23] the Whitehall II study[24] and the Health in Men Study (HIMS)[25] were used. Details of each study population are provided in the supplementary material on the journal website (Text S1) and summarised in table 1.

**Table 1:** Details of studies included in the analysis.

Study	Number (total in study)	Number CHD cases	% male	Mean (SD) age	Outcome assessment	CRP (mg/l)	CRP *+1444C>T*	*CRP*-CRP association^a^	*CRP*-CHD association
BWHHS	3549	986	0	68.8 (5.5)	Prevalent or incident fatal and non-fatal (medical records and ECG changes) angina and MI	Geometric mean (95%CI) 1.87 (1.80, 1.94) Median (IQR) 1.82 (0.84, 4.03)	CC: 1702 (48.0%) CT: 1534 (43.2%) TT: 313 (8.8%) Minor allele freq: 0.30 HWE test p = 0.21	1.23 (1.08, 1.39)	0.93 (0.72, 1.21)
Caerphilly	934	492	100	56.8 (4.5)	Prevalent or incident fatal and non-fatal (medical records and ECG changes) angina and MI	Geometric mean (95%CI) 1.63 (1.52, 1.73) Median (IQR) 1.67 (0.87, 3.43)	CC: 437 (46.8%) CT: 393 (42.1%) TT: 104 (11.1%) Minor allele freq: 0.32 HWE test p = 0.29	1.15 (0.94, 1.41)	1.20 (0.80, 1.81)
Speedwell	639	238	100	56.8 (4.3)	Prevalent or incident fatal and non-fatal (medical records and ECG changes) angina and MI	Geometric mean (95%CI) 1.31 (1.21, 1.43) Median (IQR) 1.60 (0.70, 3.50)	CC: 305 (47.7%) CT: 275 (43.1%) TT: 59 (9.2%) Minor allele freq: 0.31 HWE test p = 0.85	1.26 (0.94, 1.69)	0.72 (0.40, 1.28)
Whitehall II	5051	418	73	61.0 (6.0)	Prevalent non-fatal MI or definite angina	Geometric mean (95%CI) 1.30 (1.26, 1.34) Median (IQR) 1.22 (0.63, 2.59)	CC: 2402 (47.6%) CT: 2223 (44.0%) TT: 426 (8.4%) Minor allele freq: 0.30 HWE test p = 0.01	1.18 (1.06, 1.32)	1.18 (0.83, 1.68)
HIMS	3805	1491	100	77.1 (3.6)	For prevalent and incident cases: self-report, medical record of a hospital admission for CHD, death from CHD	Geometric mean (95%CI) 2.02 (1.95, 2.09) Median (IQR) 1.87 (1.02-3.83)	CC: 1846 (48.5%) CT: 1596 (41.9%) TT: 363(9.5%) Minor allele freq: 0.31 HWE p = 0.52	1.25 (1.12, 1.40)	1.01 (0.81, 1.26)
Casas et al Previously published pooled analysis of 6 studies	4659	985	100	‘middle-aged’	Prevalent or incident non-fatal MI (WHO criteria) assessed using same protocol across all 6 studies	Weighted mean (95%CI) in CT or CC without CVD 2.01 (1.94, 2.07)	Minor allele frequency across the 6 included studies: 0.26–0.33 5 out of 6 in HWE	1.21 (1.09, 1.34)	1.01 (0.74, 1.38)

a Ratio geometric means (95%CI) TT versus CT or CC (reference)

b Odds ratio (95%CI) TT versus CT or CC (reference)

### Assessment of CHD

Since our main analyses are concerned with the association of *CRP* genotype with CHD, and the association of genotype with later outcomes cannot be explained by reverse causation or confounding, we have used a combined outcome of prevalent CHD (i.e. cases were identified at the same time that CRP was measured and blood samples for DNA were extracted) and incident CHD (i.e. cases occurred after CRP assessment and DNA extraction) for all of our main analyses. However, we also checked that associations were similar for prevalent cases only and for incident cases only.

In the BWHHS prevalent cases were any women with self-report of a doctor diagnosis of angina or myocardial infarction, evidence in the medical record review at baseline of either of these diagnoses, or ECG-defined ischemia. Incident CHD was defined as either death from CHD (ICD10 codes I20–I25) or evidence of new angina or myocardial infarction in medical record reviews.

In Caerphilly and Speedwell prevalent CHD was defined as any man with ECG-defined ischemia or self-report of doctor diagnosed myocardial infarction or angina at baseline and incidence CHD as ECG-defined ischemia at any follow-up examination, self-report or medical record evidence of acute MI (WHO criteria), or death from CHD (ICD 9: 410–414).

In the Whitehall II study, prevalent CHD was ascertained by questionnaire items on chest pain and a physician's diagnosis of a heart attack.[26] For any participant who indicated that they had a physician diagnosis of myocardial infarction their medical records were reviewed and the myocardial infarction only confirmed if it met MONICA criteria,[27] based on electrocardiographic findings, biomarkers of myocardial necrosis and a history of chest pain in the participant's medical records. Similarly for participants whose questionnaire response indicated that they suffered with angina, this was corroborated in medical records or by abnormalities in a resting electrocardiogram (ECG), an exercise ECG, or a coronary angiogram.[26] Only cases of myocardial infarction or angina that were confirmed in medical records or by examination findings were classified as a case.

In the HIMS, prevalent CHD was defined as questionnaire-reported coronary symptoms, bypass surgery or angioplasty, or evidence from the Western Australia Linked Data System of a non-fatal CHD event. The Western Australian Linked Data System keeps a record of all inpatient admissions and all deaths in the State.[28] Incident cases were men who were free of CHD at baseline, but had a later health contact due to non-fatal or fatal CHD during the follow-up period. Relevant ICD 9 and 10 codes (see above) were used to identify prevalent and incident cases from the Western Australian Linked Data System.

### Genotyping and CRP assays

We assessed the association of +1444C>T SNP (rs1130864) in the 3′ untranslated region of *CRP* with CRP and CHD in these studies. This SNP was chosen because it has been shown to be consistently related to CRP levels and is therefore a robust instrument for exploring the causal association of CRP levels with disease outcomes.[12], [20], [29]–[32] Furthermore, this is the SNP that was used in the recent Mendelian randomization study to examine *CRP* association with CHD,[12] and it was the one *CRP* SNP that had been typed in all of our new studies. Full details of how genotyping was undertaken and CRP measured for each study are provided in the supplementary material on the journal website (Text S1).

### Assessment of potential confounders

In all cohorts weight (without shoes and in light clothing) and height were assessed using standard research procedures and used to calculate body mass index (kg/m^2^). Information on occupation (to determine socioeconomic position), smoking and physical activity were determined from standard questionnaires.

### Ethical issues

All studies had research ethics committee approvals. All participants provided informed consent to participate in the studies. In BWHHS 8 women declined consent for the biological samples to be used for genetic analyses and these women have not been genotyped.

### Statistical analysis

All analyses were conducted only on those with complete data for genotype, CRP levels and CHD (see table 1 for numbers). For each cohort Hardy-Weinberg equilibrium was tested on a contingency table of observed-versus-predicted genotypic frequencies using an exact test.[33] Natural logarithmic transformation of CRP was undertaken to ensure that residuals were approximately normally distributed in regression models.

In the main analyses we used a recessive genetic model (comparing those who were TT homozygotes for rs1130864 to C allele carriers). The main rationale for this was that the previously published Mendelian Randomization study [12] to examine this association used a recessive model and we wished to pool results from that study with those from our new studies. However, the association of rs1130864 with CRP concentration in our in-house studies and previous publications supports an additive (per T allele) genetic model. Therefore we repeated analyses for our in house study using an additive model in order to examine whether this would have changed our main conclusions.

Linear and logistic regressions were performed to examine the associations of potential confounding factors with CRP concentration and genotype. Linear regression was used to assess the association between genotype and log CRP, which is presented as the ratio of geometric mean comparing those who were TT homozygotes for rs1130864 compared to C allele carriers (or per T allele). Logistic regression was used to assess the association between log CRP and any (prevalent or incident) CHD, which is presented as the age adjusted odds ratio per doubling of CRP.

Random effects meta-analyses were used to pool results from individual studies. Each individual cohort reported in the meta-analysis undertaken by Casas et al.[12] was treated as a separate study in order to consistently model the between-study heterogeneity. Heterogeneity was assessed using the I^2^ measure that describes the percentage of total variation in the pooled estimate that is due to between study heterogeneity.[34]

Additional information on statistical analyses, including the instrumental variables analyses, and strengths and limitations of this method, are provided in the supplementary material on the journal website (Text S1).

## Results

Our analyses included 18,637 participants, of whom 4,610 were CHD cases. Across all studies 48% of participants were homozygous for the C allele, 43% were heterozygotes and 9% were homozygous for the T allele; the T allele frequency was 0.31 and the overall HWE test result (combining genotype frequencies from all of the studies in table 1) was p = 0.81. Within each study, except Whitehall II, there was no evidence for departure from HWE (see table 1). Because rs1130864 was not in the HWE in Whitehall II study, in that study the SNP was re-genotyped from 678 samples in a different laboratory and the results called by a researcher who was blind to the original results. The mismatch rate was 0.5%. In addition a repeated blood sample was obtained from 553 participants from which DNA was extracted and the SNP re-measured. The error rate was less than 1%. The departure from HWE in Whitehall II suggests that there were approximately 50 fewer T allele homozygotes observed in this sample when compared to expected frequencies assuming HWE. Our additional re-genotyping (describe above) suggests that this is most likely due to random residual (<0.5%) genotyping error, rather than to any biological selection bias or other populational inhomogeneity.

### Association of CRP levels and CRP genotype with potential confounding factors

Table 2 shows the associations of potential confounding factors with CRP levels and Table 3 shows the associations of rs1130864 with these potential confounders. In all examined cohorts higher concentration of CRP was associated with increased prevalence of obesity and smoking, as well as lower prevalence of physical activity (Table 2). In BWHHS, Caerphilly, the Whitehall II and the HIMS study, participants with higher CRP levels were more likely to be from lower socioeconomic position, but this association was not apparent in the Speedwell cohort. Genotype was not associated with these potential confounders in any of the cohorts (Table 3).

**Table 2:** Associations of potential confounding factors with CRP levels in five new cohort studies.

	Means (SD) or n (%) of potential confounding factors by thirds of the CRP distribution in 5 cohort studies			
**BWHHS N = 3549 All female**				
	Lowest 1/3	Middle 1/3	Highest 1/3	P trend
	Range: 0.16–1.13 mg/l	Range: 1.14–3.13 mg/l	Range: 3.14–112.0 mg/l	
	N = 1177	N = 1173	N = 1199	
Age mean (SD) years	68.6 (5.5)	68.9 (5.4)	68.9 (5.5)	0.14
BMI mean (SD) kg/m^2^	25.5 (3.7)	27.7 (4.6)	29.7 (5.5)	<0.001
Obese n (%)	125 (10.7)	294 (25.3)	516 (43.4)	<0.001
Low adult SEP* n (%)	392 (33.3)	440 (37.5)	520 (43.4)	<0.001
Low childhood SEP* n (%)	921 (78.2)	934 (79.6)	980 (81.7)	0.03
Current smoker n (%)	90 (7.7)	123 (10.5)	179 (14.9)	<0.001
Physical activity† n (%)	513 (44.8)	426 (38.0)	289 (25.4)	<0.001
**Caerphilly N = 934 All Male**				
	Lowest 1/3	Middle 1/3	Highest 1/3	P trend
	Range: 0.17–1.09 mg/l	Range: 1.10–2.61 mg/l	Range: 2.62–48.1 mg/l	
	N = 332	N = 308	N = 294	
Age mean (SD) years	56.0 (4.3)	56.9 (4.6)	57.6 (4.4)	<0.001
BMI mean (SD) kg/m^2^	25.4 (3.0)	27.3 (3.4)	27.1 (4.2)	<0.001
Obese n (%)	22 (6.7)	62 (20.3)	51 (18.0)	<0.001
Low adult SEP* n (%)	146 (53.2)	165 (66.5)	165 (68.5)	0.004
Low childhood SEP* n (%)	210 (85.4)	211 (87.6)	208 (92.0)	0.03
Current smoker n (%)	81 (24.4)	99 (32.3)	122 (41.6)	<0.001
Physical activity$ n (%)	124 (37.4)	101 (32.8)	90 (30.6)	0.05
**Speedwell N = 639 All Male**				
	Lowest 1/3	Middle 1/3	Highest 1/3	P trend
	Range: 0.10–0.90 mmol/l	Range: 0.91–2.50 mmol/l	Range: 2.51–28.90 mmol/l	
	N = 254	N = 216	N = 169	
Age mean (SD) years	56.4 (4.2)	57.3 (4.4)	57.0 (4.3)	0.11
BMI mean (SD) kg/m^2^	25.5 (2.8)	26.2 (3.0)	26.4 (3.2)	<0.001
Obese n (%)	10 (3.9)	20 (9.3)	19 (11.4)	0.004
Low adult SEP* n (%)	156 (61.4)	125 (57.9)	103 (61.0)	0.85
Low childhood SEP* n (%)	NA	NA	NA	NA
Current smoker n (%)	52 (20.5)	73 (33.8)	71 (42.0)	<0.001
Physical activity¶ n (%)	33 (13.0)	20 (9.3)	13 (7.7)	0.07
**Whitehall II N = 3696 Male, N = 1355 Female**				
	Lowest 1/3	Middle 1/3	Highest 1/3	P trend
	Range: 0.08–0.77 mg/L in men; 0.08–0.90 mg/L in women	Range: 0.78–1.77 mg/L in men; 0.91–2.55 in women	Range: 1.78–114.0 mg/L in men; 2.56–160.0 in women	
	N = 1680	N = 1686	N = 1685	
Age mean (SD) years	60.3 (5.8)	61.0 (6.0)	61.7 (6.0)	<0.001
BMI mean (SD) kg/m^2^	24.6 (3.3)	26.7 (3.7)	28.6 (4.7)	<0.001
Obese n (%)	98 (5.9)	292 (17.4)	536 (31.9)	<0.001
Low adult SEP‡ n (%)	113 (6.8)	139 (8.3)	159 (9.5)	0.01
Low childhood SEP* n (%)	435 (37.8)	528 (44.5)	550 (48.3)	<0.001
Current smoker n (%)	105 (6.3)	155 (9.2)	231 (13.7)	<0.001
Physical activity# n (%)	1447 (87.0)	1410 (84.9)	1388 (83.3)	0.01
**HIMS N = 4659 All male**				
	Lowest 1/3	Middle 1/3	Highest 1/3	P trend
	Range: 0.15–1.26 mg/L	Range: 1.27–2.89 mg/L	Range: 2.90–182.0 mg/L	
	N = 1276	N = 1259	N = 1270	
Age mean (SD) years	76.9 (3.6)	77.0 (3.6)	77.3 (3.7)	0.005
BMI mean (SD) kg/m^2^	25.7 (3.5)	26.7 (3.4)	27.3 (3.9)	<0.001
Obese n (%)	302 (23.7)	412 (32.7)	529 (41.7)	<0.001
Low adult SEP** n (%)	330 (25.9)	361 (28.7)	410 (32.3)	<0.001
Low childhood SEP n (%)	NA	NA	NA	
Current smoker n (%)	43 (3.3)	61 (4.8)	99 (7.8)	<0.001
Physical activity†† n (%)	396 (31.0)	345 (27.4)	304 (23.9)	0.01

CRP: C-reactive protein; SD: standard deviation; BMI: body mass index; SEP: Socioeconomic position; n: number

NA: data on childhood SEP not available for Speedwell and the HIMS participants

* Defined as manual occupational social class according to British Registrar General's Classification

† Defined as at least 2 hours per week of moderate of vigorous exercise

$ Defined as highest third of the distribution of total energy expenditure derived from the Minnesota Leisure Time Physical Activity

¶ Defined as participating in regular swimming or morning exercises

‡ Defined as clerical (lowest) employment grade

# Defined as non-sedentary

** Defined as socioeconomic disadvantage based on a score of less than 1,000 on the Australian 1996 index of disadvantage (http://www.facsia.gov.au/research/prp08/PRP_No_08.pdf)

†† Defined as two or more episodes of vigorous activity per week

**Table 3:** Associations of potential confounding factors with CRP gene (+1444C>T) in five new cohort studies.

	Means (SD) or n (%) of potential confounding factors by genotype in BWHHS		
	N = 3549 All female		
**BWHHS N = 3549 All female**			
	CC or CT	TT	p
	N = 3236	N = 313	
Age mean (SD) years	68.8 (5.5)	68.9 (5.4)	0.61
BMI mean (SD) kg/m^2^	27.7 (4.9)	27.6 (5.3)	0.74
Obese n (%)	855 (26.6)	80 (25.8)	0.76
Low adult SEP* n (%)	1236 (38.2)	116 (37.1)	0.69
Low childhood SEP* n (%)	2587 (79.9)	248 (79.2)	0.76
Current smoker n (%)	358 (11.1)	34 (10.9)	0.91
Physical activity† n (%)	1133 (36.5)	95 (32.1)	0.14
**Caerphilly N = 934 All Male**			
	CC or CT	TT	p
	N = 830	N = 104	
Age mean (SD) years	56.8 (4.5)	57.0 (4.6)	0.71
BMI mean (SD) kg/m^2^	26.6 (3.7)	26.4 (3.0)	0.63
Obese n (%)	126 (15.4)	9 (8.8)	0.08
Low adult SEP* n (%)	430 (64.3)	46 (57.5)	0.24
Low childhood SEP* n (%)	564 (89.0)	65 (82.3)	0.09
Current smoker n (%)	270 (32.6)	32 (30.8)	0.71
Physical activity$ n (%)	278 (33.5)	37 (35.6)	0.67
**Speedwell N = 639 All Male**			
	CC or CT	TT	p
	N = 580	N = 59	
Age mean (SD) years	56.8 (4.3)	57.2 (4.4)	0.52
BMI mean (SD) kg/m^2^	25.8 (3.0)	26.2 (3.6)	0.32
Obese n (%)	41 (7.1)	8 (13.8)	0.07
Low adult SEP* n (%)	349 (60.2)	35 (59.3)	0.90
Low childhood SEP n (%)	NA	NA	NA
Current smoker n (%)	179 (31.0)	17 (28.8)	0.75
Physical activity¶ n (%)	57 (9.8)	9 (15.3)	0.20
**Whitehall II N = 3696 Male; N = 1355 Female**			
	CC or CT	TT	p
	N = 4625	N = 426	
Age mean (SD) years	61.0 (5.9)	60.6 (6.0)	0.18
BMI mean (SD) kg/m^2^	26.7 (4.3)	26.6 (4.1)	0.76
Obese n (%)	849 (18.4)	77 (18.2)	0.89
Low adult SEP‡ n (%)	376 (8.2)	35 (8.3)	0.96
Low childhood SEP* n (%)	1398 (43.8)	115 (40.6)	0.31
Current smoker n (%)	457 (9.9)	34 (8.0)	0.21
Physical activity# n (%)	3889 (85.1)	356 (84.2)	0.59
**HIMS N = 4659 All male**			
	CC or CT	TT	p
	N = 3442	N = 363	
Age mean (SD) years	77.1 (3.6)	76.9 (3.6)	0.30
BMI mean (SD) kg/m^2^	26.6 (3.6)	26.7 (3.7)	0.45
Obese n (%)	1126 (32.7)	117 (32.2)	0.85
Low adult SEP‡ n (%)	997 (29.0)	104 (28.7)	0.85
Low childhood SEP* n (%)	NA	NA	
Current smoker n (%)	184 (5.3)	19 (5.2)	0.93
Physical activity# n (%)	950 (27.6)	95 (26.2)	0.56

CRP: C-reactive protein; SD: standard deviation; BMI: body mass index; SEP: Socioeconomic position; n: number

NA: data on childhood SEP not available for Speedwell the HIMS participants

* Defined as manual occupational social class according to British Registrar General's Classification

† Defined as at least 2 hours per week of moderate of vigorous exercise

$ Defined as highest third of the distribution of total energy expenditure derived from the Minnesota Leisure Time Physical Activity

¶ Defined as participating in regular swimming or morning exercises

‡ Defined as clerical (lowest) employment grade

# Defined as non-sedentary

** Defined as socioeconomic disadvantage based on a score of less than 1,000 on the Australian 1996 index of disadvantage (http://www.facsia.gov.au/research/prp08/PRP_No_08.pdf)

†† Defined as two or more episodes of vigorous activity per week

### Association of CRP levels and CHD

**Figure 1:**
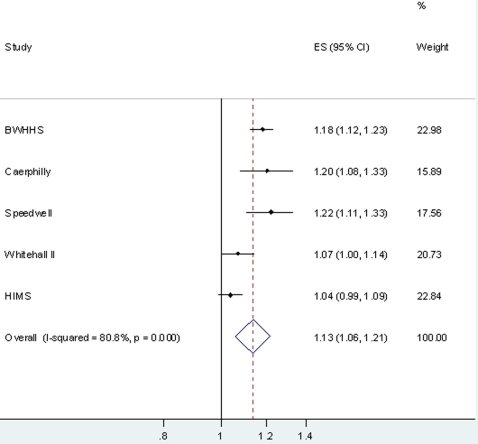
Pooled age and sex adjusted odds ratio (95% confidence interval) of CHD per doubling of CRP levels. Results from 5 cohort studies of 13, 978 participants of whom 3,625 were CHD cases (prevalent or incident).

**Figure 2:**
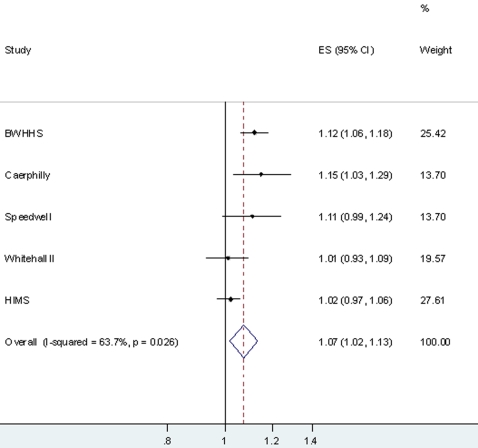
Pooled age, sex and confounder adjusted odds ratio (95% confidence interval) of CHD per doubling of CRP levels. Results from 5 cohort studies of 13, 978 participants of whom 3,625 were CHD cases (prevalent or incident). Confounders included in multivariable models = age, sex, smoking, body mass index, physical activity, socioeconomic position.

Table 4 shows the association between circulating CRP and CHD. Associations were of a similar magnitude for prevalent CHD (CRP levels measured at same time as history of CHD ascertained) and incident CHD (CRP levels measured before new cases of CHD) in each cohort, and with a combined outcome of prevalent and incident CHD. With adjustment for confounders (body mass index, smoking, socioeconomic position and physical activity) the positive age and sex adjusted associations attenuated towards the null in each cohort, though positive associations remained in BWHHS, Caerphilly and Speedwell. When results from all five cohorts were pooled in a meta-analysis a doubling of CRP was associated with an odds ratio of CHD of 1.13 (95%CI: 1.06, 1.21) in age and sex adjusted models (Figure 1) and of 1.07 (95%CI: 1.02, 1.13) in age, sex and confounder adjusted models (Figure 2). There was evidence of heterogeneity between studies in both of these meta-analyses (I^2^ = 80.8% in the age and sex adjusted analyses and 63.0% in age, sex and confounder adjusted analyses). None of the percentage of participants who were male in each study, mean age of study participants, the percentage of cases that were incident in each study or the percentage of cases that were hard CHD outcomes (acute MI or death from CHD) explained between study heterogeneity in either the age adjusted or the full confounder adjusted meta-analyses (all p-values >0.2).

**Table 4:** Association of CRP with CHD in five new cohort studies. Total number included across all 5 cohorts 13, 978, of whom 3,625 had either prevalent or incident CHD

	Associations with Prevalent CHD cases*			Associations with Incident CHD cases*			Associations with Prevalent and Incident CHD cases*		
	N cases	Age and sex† adjusted OR (95%CI) per doubling of CRP	Age, sex† and confounder$ adjusted OR (95%CI) per doubling of CRP	N cases	Age and sex† adjusted OR (95%CI) per doubling of CRP	Age, sex† and confounder$ adjusted OR (95%CI) per doubling of CRP	N cases	Age and sex† adjusted OR (95%CI) per doubling of CRP	Age, sex* and confounder$ adjusted OR (95%CI) per doubling of CRP
BWHHS	850	1.18 (1.11, 1.25)	1.15 (1.08, 1.22)	136	1.18 (1.07, 1.30)	1.14 (1.03, 1.26)	986	1.18 (1.12, 1.23)	1.12 (1.06, 1.18)
Caerphilly	182	1.14 (1.02, 1.27)	1.07 (0.95, 1.21)	310	1.20 (1.09, 1.33)	1.12 (0.99, 1.28)	492	1.22 (1.11, 1.33)	1.11 (0.99, 1.24)
Speedwell	123	1.13 (0.98, 1.33)	1.12 (0.96, 1.32)	115	1.22 (1.07, 1.38)	1.17 (1.02, 1.34)	238	1.20 (1.08, 1.33)	1.15 (1.03, 1.29)
Whitehall II	418	1.07 (1.00, 1.14)	1.01 (0.93, 1.09)	-	-	-	418	1.07 (1.00, 1.14)	1.01 (0.93, 1.09)
HIMS	860	1.02 (0.97, 1.08)	1.00 (0.95, 1.05)	631	1.09 (1.03, 1.16)	1.07 (1.01, 1.13)	1491	1.04 (0.99, 1.09)	1.02 (0.97, 1.06)

* Prevalent cases are cases of CHD that were determined at the same time that CRP levels were assessed (i.e. these associations are cross-sectional); Incident cases were new occurrences of CHD that occurred after the baseline assessment of CRP. In the analyses with incident cases only, those with prevalent CHD were excluded.

† BWHHS includes women only; Caerphilly, Speedwell & HIMS include men only; Whitehall II includes women and men–the association of CRP with CHD was the same in women and men (all p-values for interaction >0.5) and results are presented for women and men combined with adjustment for sex

$ Adjusted for body mass index, smoking, physical activity and socioeconomic position in addition to age and sex.

For this study Whitehall II only has prevalent cases of CHD (i.e. individuals who already had CHD at the time that blood samples were taken for CRP levels and *CRP* genotype)

### Association of CRP genotype with CRP levels and with CHD events

**Figure 3:**
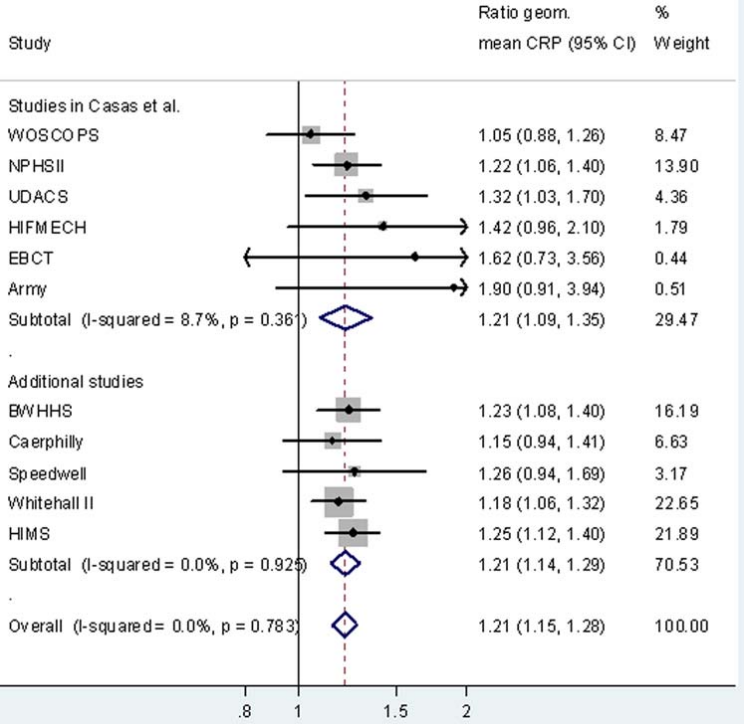
Association of *CRP* rs1130864 with CRP levels. Results are the geometric mean (95%CI) of CRP levels comparing individuals with TT genotype to those with the CT or CC genotype (reference group). The results are from studies of 18, 637 participants of whom 4,610 were CHD cases (prevalent or incident).

Genotype (rs1130864) was associated with circulating CRP in all cohorts (Table 1), with the pooled ratio of geometric means comparing individuals with the TT genotype to those with the CT/CC genotype being 1.21 (95%CI: 1.15, 1.28) (Figure 3). There was no detectable between-study heterogeneity in this analysis (I^2^ = 0%, p = 0.90). The pooled (for our 5 in-house studies only) per T allele ratio of geometric means was 1.13 (95%CI: 1.10, 1.16), with no detectable between-study heterogeneity (I^2^ = 0%, p = 0.91). Despite the robust associations of rs1130864 with circulating CRP this variant explained only a small proportion of its variation: within BWHHS (women only) it explained 0.8%; within Caerphilly (men only) 0.4%; within Speedwell (men only) 0.6%; within Whitehall II (73% men) 0.4% and within HIMS (men only) 0.7%.

**Figure 4:**
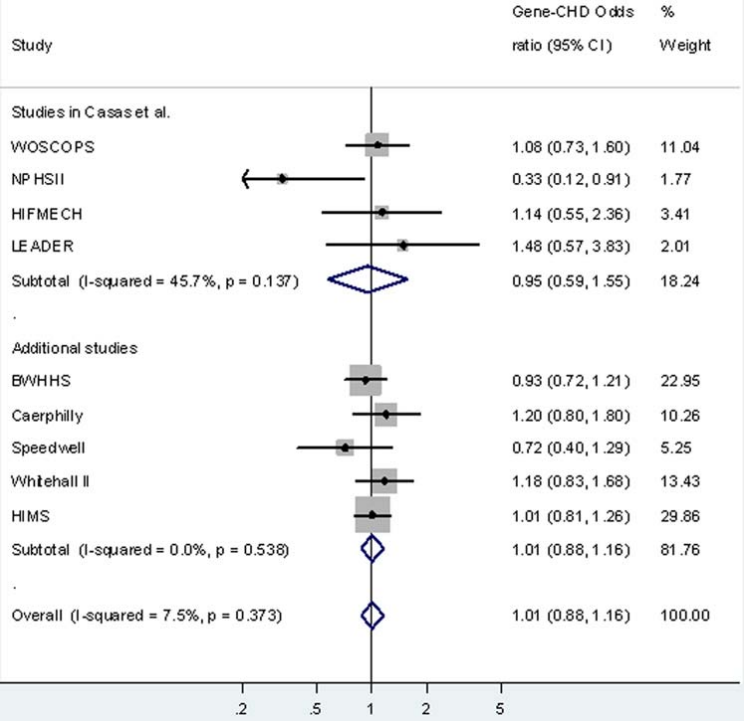
Association of *CRP* rs1130864 with CHD. Results are the odds ratio (95%CI) of having CHD comparing individuals with TT genotype to those with the CT or CC genotype (reference group). The results are from cohort studies of 18, 637 participants of whom 4,610 were CHD cases (prevalent or incident).

There was no strong evidence of an association between rs1130864 and CHD in any of the studies. Table 1 presents associations, by study, of genotype with a combined outcome of prevalent and incident CHD; results were the same when associations were examined for prevalent cases alone and for incident cases alone. The pooled odds ratio of CHD comparing individuals with the TT genotype to those with the CT/CC genotype was 1.01 (95%CI: 0.88, 1.16) (Figure 4). There was minimal between-study heterogeneity in this analysis (I^2^ = 7.5%, p = 0.62). The pooled (for our 5 in-house studies only) per T allele odds ratio of CHD was 0.96 (95%CI: 0.90, 1.03), with no detectable between-study heterogeneity in this analysis (I^2^ = 0%, p = 0.99).

There was no evidence of correlation between rs1130864-CRP concentration and rs1130864-CHD estimates from separate studies on either a scatter plot (not shown) or as measured by the correlation coefficient of −0.089 (p* = *0.83), justifying our assumption of no correlation in our use of Fieller's theorem for estimating the confidence interval for the instrumental variables analysis (see website supplementary material for methods: Text S1). Combining the rs1130864-CRP and rs1130864-CHD summary estimates gave an instrumental variable estimate (see website supplementary material for methods: Text S1) of the odds ratio of CHD for a doubling of CRP concentration of 1.04 (95% CI: 0.61, 1.80). Findings were similar, but with wider confidence intervals, when incident cases only were included in the instrumental variables analyses.

## Discussion

Meta-analyses of prospective observational studies have demonstrated a positive association between circulating CRP and CHD risk.[35]–[37] However, it remains unclear whether this association is causal or explained by confounding factors or reverse causality, or even underestimated as a result of attenuation by errors.[7], [38] The best method to establish causality for this association would be by randomization to an intervention that altered CRP levels but did not affect any other cardiovascular risk factors. To our knowledge no such trials have been conducted. The use of genetic variants as instrumental variables to determine the causal association between circulating CRP and CHD provides an alternative to such a randomized controlled trial, with the advantage that this approach can be performed in existing datasets and may be more generalisable than a randomized controlled trial.[8], [9], [10] It has also been suggested that CRP genetic profiling might have clinical utility in predicting CHD risk.[20]

In this study of 4,610 cases (the study with the largest numbers to date to examine the association of variation in *CRP* with CHD), we present evidence that higher circulating CRP level is unlikely to be an important causal risk factor for CHD. We found no association of a genetic variant (rs1130864) in *CRP* with CHD events, despite this variant being consistently associated with circulating CRP. Using rs1130864 to explore the causal association of CRP levels with CHD risk is valid since individuals who are homozygous for the T allele (TT genotype) will have experienced on average higher levels of circulating CRP over their lifetime than other individuals (CT or CC genotype),[39] but potential confounding factors will be evenly distributed between these two groups of individuals (TT versus CT or CC), as demonstrated for all our cohorts in table 3. Thus, the association of rs1130864 with CHD cannot be influenced by reverse causality, attenuation by errors or confounding. [8], [9], [10], [40] In this respect, our analysis of the association of rs1130864 with CHD can be compared to a randomised controlled trial of individuals who have been randomly allocated (or not) to a 21% higher CRP level on average across their lives, given that our pooled ratio of geometric means of CRP by genotype was 1.21.

The assumptions underlying the Mendelian randomization approach is that the genetic variant is associated with the modifiable risk factor (circulating CRP levels in this example) and that it is not related to the outcome of interest (CHD) other than through its association with the modifiable risk factor (i.e. there are no confounding factors relating genotype to CHD and genotype is not related to CHD through other pathways).[8], [9], [10] The rs1130864 SNP has been consistently shown to be associated with circulating CRP levels in males and females of European origin,[12], [20], [29]–[31] a finding that we have replicated here. Furthermore we have previously shown that the rs1130864 SNP is associated with a shift in the whole distribution of CRP levels and not an increase, for example, only at the higher end or a decrease only at the lower end of the CRP distribution.[41] This whole-population shift in CRP levels should clearly be related to a whole-population shift in CHD risk if CRP were causally related to CHD and therefore our null finding is unlikely to be explained by missing individuals with CRP levels above or below a given threshold in relation to genotype.

There are strong theoretical bases for believing that *CRP* genotype will not be related to socioeconomic and behavioural confounding factors that tend to distort observational epidemiological studies of this association,[8], [9], [10], [42] and we have empirically demonstrated here that whilst circulating CRP is related to these potential confounding factors, genotype is not (Tables 2 and 3).

The variant that we have used in this study is in close linkage disequilibrium (LD) with variation within a transcription factor binding site located 5′ of *CRP* that is associated with circulating concentrations of CRP and thought to be functional.[43]–[45] It is unlikely that this functional variant, or one in close LD with it, will have pleiotropic effects. It is possible that another variant in another gene near *CRP* is in LD with rs1130864 and provides another pathway to CHD. To explain our null result in the context of circulating CRP being truly causally related to CHD this alternative pathway would have to result in a decrease in CHD risk by a magnitude that exactly reversed the posited causal influence of circulating CRP on CHD risk. However, rs1130864 exhibits no major LD (correlation R^2^>0.2) with SNPs in any other gene in its genomic region, except with a nearby gene (ENSG00000196401) identified in Ensembl and HapMap R^2^ = 0.8, but not shown in Entrez. For lower levels of LD (i.e. with R^2^< = 0.2), the effect of any confounding gene on CHD would have to be so large as to be implausible (no such gene has been found in any CHD genome wide studies to date [46]–[48]). Variant ENSG00000196401 (XR 017178.1) shows sequence similarity to ribosomal protein L27 (LOC646446) mRNA, which is widely expressed. However, there is no strong evidence that ribosomal proteins in general, ribosomal protein L27 specifically, nor ENSG00000196401 gene are associated with CHD risk. Of note, none of the recent genome-wide association studies identified variants in ENSG00000196401 as being related to CHD risk.[46]–[48] It is therefore unlikely that the variant we have examined here (rs1130864) is linked via alternative molecular genetic pathways to CHD risk in such a way that these alternative pathways completely counter balance an important circulating CRP causal effect on CHD.

A number of SNPs related to CRP levels have been identified in *CRP* and we would have had greater statistical power for our instrumental variables analyses had we constructed haplotypes using several SNPs as in one of our previous papers.[32] However, we were limited here to using a SNP that was typed in all studies included in the analyses. This should not have biased our results, which are consistent with other studies showing that genetic variation in several SNPs in *CRP* are not associated with CHD in the way predicted by their association with CRP levels (see introduction and further discussion below).

Within each of our cohort studies participants are described as being ‘white’, ‘Caucasian’, or of ‘European’ origin and the consistency of association between rs1130864 and both CRP levels and CHD events across our studies suggests that population stratification is unlikely to have importantly confounded our genetic association results. Developmental canalisation (the process by which target receptors or organs develop differently in response to varying levels of the exposure of interest during key developmental periods) might limit the Mendelian randomization process. The extent to which this occurs with modest effects such as the differences in CRP level by genotype is unclear.[10]

For our main analyses we combined incident and prevalent cases. In conventional observational epidemiology incident cases are important for making causal inference and minimising any bias due to reverse causality. However, in genetic association studies reverse causality is not possible and survivor bias very unlikely.[8], [9], [10] Repeating our analyses with incident cases only did not substantively alter any of the point estimates for any of our analyses.

There was important heterogeneity between individual studies in the association of circulating CRP with CHD that was not explained by differences in the distribution of sex, age or the proportion of incident cases between studies. Adjustment for potential confounding factors reduced heterogeneity but some remained even in the confounder adjusted analyses, suggesting that residual confounding in some studies might explain this heterogeneity. Since our Mendelian randomization study relates the proportion of circulating CRP that is explained by rs1130864 to CHD risk, and there was no between study heterogeneity in the association of this genetic variant with CRP levels or with CHD, our Mendelian randomization results are unaffected by between study heterogeneity in the observational association of CRP with CHD.

Our instrumental variables analysis uses the proportion of the variation in CRP that is explained by rs1130864 to provide an estimate of causal effect that is not biased by confounding, reverse causality or attenuation by errors. However, the advantage of this approach of being less biased than a conventional multivariable regression analysis comes at the cost of reduced precision. Although the point estimate of the odds ratio per doubling of CRP from the instrumental variables analysis was virtually null (1.04), the 95% confidence interval (0.61, 1.80) includes the observational association, and the association found in the most recent meta-analysis of observational studies.[37] The confidence interval for the instrumental variables analysis is much wider than the confidence interval from the observational analyses presented here or in previous meta-analyses.[37] An alternative to the instrumental variable analyses in Mendelian randomization studies is to compare the observed genetic-disease association to that expected from the best observational studies, as done recently by Casas et al.[12] Whilst this approach appears to provide more precise estimates of the causal association of CRP with CHD and does not require that all studies included in the analyses have measurements of all three of CRP levels, *CRP* genotype and CHD, the level of precision is in fact spurious since it does not fully account for the relatively small proportion of total variation in CPR accounted for by genotype and does not include uncertainty from all analyses.[10]

The fact that rs1130864 explains less than 1% of the variation in CRP within each of our studies, whilst affecting statistical precision is unlikely to result in bias. As noted previously,[8], [9], [10] many medications that are used in randomised controlled trials to determine causality explain a similarly small proportion of variation in the potentially causal risk factor, but with adequate sample sizes (sometimes, as in our Mendelian randomization study presented here, obtained only through meta-analysis of data from a number of trials) provide precise and valid estimates of the causal effect on clinical endpoints.

For example, blood pressure lowering therapies explain ∼2% of the variation in blood pressure, and in participants who are randomised to either active blood pressure lowering therapy or control in randomised controlled trials there will be many other environmental and genetic factors that influence variation in blood pressure. Nonetheless, an adequately powered randomised trial of the effect of blood pressure medication on stroke (or other cardiovascular outcomes) is, rightly, accepted as unbiased evidence of the causal effect of blood pressure on stroke risk.[9], [10]

With respect to other Mendelian randomization studies, single SNPs that have been shown to be robustly associated with low density lipoprotein cholesterol (LDLc), and that explain less than 1% of the variation in circulating LDLc, are robustly associated with CHD, with the magnitude of this association being somewhat larger than that predicted from the causal randomised controlled trial evidence relating statins (which reduce LDLc) to CHD.[49], [50] It has been suggested that the somewhat stronger effects with these genetic variants relates to the fact that the randomised difference in LDLc occurring as a result of genetic variants is life-long, whereas that occurring as a result of statins is from mid-adult life only.[51], [52] Similarly, we have recently shown that a single SNP in *FTO* (which again explains less than 1% of variation in body mass index or total fat mass) is associated with a wide range of vascular and metabolic outcomes, including fasting glucose, insulin and lipids, with magnitudes of association that are the same as would be predicted from the association of *FTO* with BMI and of BMI with these outcomes in observational studies and trials of weight reduction.[53] It would seem odd to us that the Mendelian randomization approach provides valid causal estimates in examples where causation is not controversial (i.e. the effect of LDLc on CHD and BMI on fasting glucose) but is selectively biased in more debatable areas such as that assessed here and in the companion paper[21] (i.e. the association of CRP with atherosclerosis and CHD risk).

Our Mendelian randomization findings are consistent with other studies that have used variants in *CRP* to explore the causal association of CRP levels with continuously measured vascular and metabolic traits and which suggest that CRP is not causally related to blood pressure, metabolic syndrome components or carotid intima media thickness.[2], [19], [32], [54] As discussed in the introduction a number of other studies, which have not directly employed Mendelian randomization approaches and have included between 210 to 1062 CHD cases, have also found genetic variants within the *CRP* gene to be unrelated to prevalent and incident CHD events, despite these variants being associated with CRP levels,[13]–[17] or have found associations in the opposite direction to what one would anticipate if higher CRP levels were causally associated with increased CHD risk,[13] or associations only in very specific subgroups.[18], [19] Whilst such subgroup analyses might represent true differences in the effect of *CRP* or CHD, in general subgroup analyses fail to replicate and should be treated with caution until replicated in other independent studies. Furthermore, none of the large genome wide association studies of CHD have found variation in *CRP* to be robustly associated with CHD.[46]–[48] By contrast variants associated with established risk factors for CHD (e.g. LDLc) are identified in these genome wide association studies CHD. Taking these findings together with our own results–the largest study to date (N = 4160 cases; four times the largest previous published study) to relate variation in *CRP* to CHD–there does not appear to be any strong evidence for an association of *CRP* with CHD events, despite its association with CRP levels. These findings together would suggest that variation in circulating CRP level is not importantly causally related to CHD risk and that genetic profiling of *CRP* is unlikely to be useful in the prediction of CHD. However, we acknowledge that a very large genetic (Mendelian randomization) study is required to definitively demonstrate whether there is no causal association of CRP with CHD. A newly established collaboration that will include at least 30,000 CHD events will over the coming years be able to provide this definitive answer.[55]

In a companion paper we examined the association of 3 tag SNPs [+1444T>C (rs1130864); +2303G>A (rs1205) and +4899T>G (rs 3093077)] in the *CRP* gene with serum CRP and carotid intima-media thickness (CIMT) in one of the studies included in this paper (the Whitehall II Study).[21] In that study there was no independent association of CRP concentration with CIMT once potential confounding factors had been taken into account and no evidence from analyses using haplotypes in *CRP* as instrumental variables that CRP concentration is causally related to CIMT.[21] As CIMT is a marker of atherosclerosis, that paper,[21] together with other studies finding no association of genetic variation in *CRP* with CIMT,[19], [54] fail to support CRP as a causal factor for atherosclerosis.

Whilst our findings, together with those of several other studies described above, suggest that circulating levels of CRP are not importantly causally related to the development of atherosclerosis or CHD, an effect of CRP on case-fatality in those with CHD, is possible. Such an effect is implicated by findings from rodent models of beneficial effects of post-myocardial infarction CRP lowering,[56] but to our knowledge has not be demonstrated in humans.

Our findings, together with those from a number of other studies examining the association of genetic variation in *CRP* with CIMT and CHD,[12]–[19], [21], [54] and with findings from genome wide association studies of CHD,[46]–[48] suggest that circulating CRP does not have an important causal association with CHD. In our study the instrumental variables result (an odds ratio of 1.04 per doubling of CRP) is our best estimate of causal effect. However, very large genetic association studies are required to provide a precise estimate of this association and rule out possible modest causal effects.[55]

**Data Access:** DAL had full access to all of the data from the BWHHS, Caerphilly and Speedwell studies used in this paper; MK and MGM had full access to all of the data from the Whitehall II study and NW and LJP had full access to all of the data from HIMS. These individuals take responsibility for the integrity of the data and the accuracy of the data analysis.

## Supporting Information

Text S1 (0.04 MB DOC)
