# Supplementary material for: The Association of C-Reactive Protein and CRP Genotype with Coronary Heart Disease: Findings from Five Studies with 4,610 Cases amongst 18,637 Participants
Source: PLoS One. 2008 Aug 20;3(8):e3011. doi: 10.1371/journal.pone.0003011 (PMC2507759; doi:10.1371/journal.pone.0003011)
Supplement: Text S1 — (0.04 MB DOC) [file pone.0003011.s001.doc]

Supplementary text S1

The association of C-reactive protein and *CRP* genotype with coronary heart disease: Findings from five studies with 4,610 cases amongst 18,637 participants

Lawlor DA et al.

*Study populations*

BWHHS consists of 4,286 women who were aged 60 to 79 years at their baseline assessment (1999-2001) and who were randomly selected from 23 British towns. These women were interviewed, examined (including anthropometry, blood-pressure, 12-lead ECG and lung function tests), provided fasting blood samples, completed medical questionnaires and had detailed reviews of their medical records at their baseline assessment. They have been followed-up over a median of 4.7 years by flagging with the National Health Service (NHS) central register for mortality data and reviews of their medical records every two years. Prevalent CHD in this cohort refers to women with established CHD at the 1999-2001 baseline assessment; incident cases are new events (defined below) occurring in those free of CHD at baseline during the follow-up period.

Phase I of the Caerphilly study included 2,512 men aged 45-59 years from Caerphilly in Wales who underwent a baseline examination between1979-1983. In phase II (1984-1988), men who took part in phase I were re-contacted, and men who had moved into the study area were invited to participate (total n = 2,398). These men completed a standard medical history and a physical examination (including weight, height, blood pressure and 12-lead ECG). In the current analyses phase II (age range 47-67 years) is used as the baseline since CRP levels used here were measured on a blood sample collected at this phase. These men have been followed-up over the ensuing 20 years with repeat examinations every 4-5 years. Prevalent CHD in this cohort refers to men with established CHD at the phase II assessment; incident cases are new events (defined below) occurring in those free of CHD at phase II during the follow-up period.

The Speedwell study was a prospective cohort study of middle aged men residing in Speedwell, an urban district of the city of Bristol. 2,348 men aged 45-59 years on 1st September 1978 from two large group general practices in Speedwell participated in the baseline measurements in 1979-82. Men attending the research clinic completed a standard medical history and a physical examination (including weight, height, blood pressure and 12-lead ECG). These men have been seen on four further follow-up examinations with CRP being measured from a blood sample taken at phase II (age range 48-66 years). Prevalent CHD in this cohort refers to men with established CHD at the phase II assessment; incident cases are new events (defined below) occurring in those free of CHD at phase II during the follow-up period.

The Whitehall II study is a prospective cohort study of London-based civil servants. 6,895 men and 3,413 women aged 35 to 55 participated in the baseline screening in 1985-1988. Since then data collection has been repeated 7 times, the baseline and odd-numbered phases involving a clinical screening and self-administered questionnaire and the even-numbered phases questionnaire only. In 2003-2004 (phase 7), 6,062 participants were successfully genotyped for rs1130864. We excluded non-white subjects (n=488) and those with missing data on CRP concentration (n=523); thus, the final cohort for this study comprised 5,051 (3696 men, 1355 women) participants aged 50 to 74 years when samples were taken for genotyping at phase 7. For this cohort all CHD cases are prevalent – i.e. they were individuals with established CHD at the time of CRP measurement and genotyping during phase 7.

The Health in Men Study (HIMS) is based on a cohort of 12,203 male residents of Perth, Western Australia, who, as part of a population-based randomised controlled trial, underwent ultrasound screening for abdominal aortic aneurysm between 1996 and 1998, at which time they were aged between 65 and 83 years.^1^ Of ~9,000 surviving men, 4,263 attended a recall survey conducted between October 2001 and August 2004, representing 47% of surviving men. Smoking, physical activity and experience of coronary symptoms and interventions (bypass surgery and angioplasty) were assessed by self-report questionnaire at both the initial screening examination and the 2001-04 follow-up survey. Blood was collected for biological samples, including DNA extraction and CRP assays at the 2001-04 follow-up survey only. Men were aged 70-87 years at the follow-up survey when genotype and CRP levels were assessed. Prevalent CHD in this cohort refers to men with established CHD at the 2001-04 assessment; incident cases are new events occurring in those free of CHD at the 2001-04 assessment during the follow-up period, which extended to December 2006.

# Details of genotyping and measurement of CRP

For all three of the BWHHS, Caerphilly and Speedwell, DNA was extracted from K-EDTA whole blood samples by salting out procedure.^2^ All genotyping was performed by KBioscience ([**http://www.kbioscience.co.uk**](http://www.kbioscience.co.uk/)). SNPs were genotyped using the KASPar chemistry, which is a competitive allele specific PCR SNP genotyping system using FRET quencher cassette oligos ([**http://www.kbioscience.co.uk/genotyping/genotyping-chemistry.htm**](http://www.kbioscience.co.uk/genotyping/genotyping-chemistry.htm)). Blind duplicates, plate-identifying blank wells and Hardy-Weinberg equilibrium tests were used as quality control tests. CRP was measured in citrated plasma for BWHHS and Caerphilly and in serum for Speedwell.^3;4^ In all three cohorts CRP concentration was assayed by a high-sensitivity immunonephelometric assay on a ProSpec protein analyser (Dade-Behring, Milton Keynes, UK) in the University of Glasgow laboratory (GDOL, AR); intra- and inter-assay coefficients of variation were 4.7% and 8.3% respectively.

In the Whitehall II study, DNA was extracted from blood samples obtained at phase 7 using magnetic beads technology (Geneservice Ltd, Cambridge). rs1130864 was genotyped using the ABI Prism 7900HT Sequence Detection System for both PCR and allelic discrimination (Applied Biosystems, Foster City, CA) and Assays By Design from Applied Biosystems under standard conditions. Genotype calling was done manually from the PCR run component tab. Because rs1130864 was not in the Hardy Weinberg Equilibrium (HWE test p=0.003), the SNP was re-genotyped from 678 samples in a different laboratory and the results called by a researcher who was blind to the original results. The mismatch rate was 0.5%. In addition a repeated blood sample was obtained from 553 participants from which DNA was extracted and the SNP re-measured. The error rate was less than 1%. The departure from HWE in Whitehall II suggests that there were approximately 50 fewer T allele homozygotes observed in this sample when compared to expected frequencies assuming HWE. Further exploration (describe above) suggested that this was most likely due to random residual (<0.5%) genotyping error, rather than to any biological selection bias or other populational inhomogeneity. Since all CHD cases in this study were prevalent it is possible that there has been selective loss of T allele homozygotes that could result in survivor bias (i.e. if those homozygous for the T allele are at greater risk of fatal CHD events). However, there was no strong statistical evidence of deviation from HWE when genotype frequencies from all of our cohorts were combined, and there was no strong statistical evidence that the association of rs1130864 with either CRP concentration or CHD differed between Whitehall II and our other cohort studies (see results in main paper). Furthermore within other studies that included both prevalent and incident cases of CHD the magnitude of association of rs1130864 with prevalent cases was not different than its association with incident cases.

In Whitehall II CRP was measured in serum,^5^ in the same laboratory and using the same methods (with same coefficients of variation) as that used in all three Bristol studies.

In the HIMS, rs1130864 was analysed using TaqMan probes and PRC primers that were run through the Assay-by-Design service (Applied Biosystems). Blind duplicates, plate-identifying blank wells and Hardy-Weinberg equilibrium tests were used as quality control tests. CRP was measured in serum by a high-sensitivity assay, with the use of the particle enhanced immunonephelometry system on the BNII analyzer (Dade Behring, Milton Keynes, UK); intra- inter-assay coefficients of variance 4-7%.

*Additional information on statistical analyses*

We assumed there was a linear relationship between log(CRP) and the log-odds of CHD. This assumption was tested by two methods: (i) splitting log(CRP) into 5ths of its distribution and computing a likelihood ratio test comparing a model with 4 indicator variables derived from the 5 categories of log(CRP) (non-linear model) to a model with the 5 categories entered as a single ordered categorical variable (linear model) and (ii) computing a likelihood ratio test comparing a model that contained a second-order and first-order term for log(CRP) (a quadratic model) to one containing only the first-order term (linear model). For all individual cohorts except HIMS there was no strong statistical evidence for departure from a linear association (all p-values > 0.3). In HIMS there was statistical evidence that the association of log(CRP) with CHD was quadratic rather than linear (p = 0.007).

In the study by Casa et al.^6^ the results were presented as the association between the genotype and CHD, which may be small not only if there is little causal effect of CRP levels on CHD, but also if genotype explains little of the variation in CRP levels. This statistical approach can be easily misinterpreted as implying that the causal effect of interest is more precisely estimated than the epidemiological association of interest (CRP levels with CHD) really is. We prefer to present estimates for the causal effect of epidemiological interest, namely that between CRP levels and CHD derived from an instrumental variables analysis, which is less prone to spuriously narrow confidence intervals.^7;8^

We calculated the instrumental variables estimate of the log odds ratio of CHD per unit increase in log(CRP) using a ratio (Wald) estimate^7^ by dividing the summary estimate of the gene-CHD log odds ratio by the summary estimate of the mean difference in log-CRP between the two genotype categories. A confidence interval for this ratio was obtained using Fieller’s theorem,^9;10^ assuming that there was no correlation between these two summary estimates. However, we did assess whether there was evidence of such correlation by plotting the gene-CHD log odds ratio against the mean difference in log-CRP and calculating an unweighted product-moment correlation coefficient between the two.^10^ Finally, the estimate and confidence interval for the instrumental variable ratio was converted to the odds ratio of CHD for a doubling of CRP by multiplying by 2/*e* and exponentiating.

Whilst we believe it is important in Mendelian randomization studies to provide effect estimates (together with their confidence intervals) derived from relating the proportion of the risk factor of interest (e.g. CRP levels) to the outcome, some additional considerations are required when the outcome is binary, as in the case here. The method used to compute the instrumental variables estimate of the effect of CRP levels on CHD is an approximation that is not strictly correct for nonlinear models such as the logistic-linear dependence of the probability of CHD on log(CRP).^11^ We have demonstrated that there is no strong evidence of departure from linearity in the association of log(CRP) with CHD in all except one of the cohorts, and exclusion of this cohort from our analyses does not materially alter our results. However, a truly unbiased estimate could only be obtained if additional assumptions are made, including assumptions that it is impossible to check about the distribution of the unmeasured confounders.^11^ That said, the methods that we have used do provide a valid test of the null hypothesis of no effect of CRP on CHD even when these additional assumptions and that of linearity are not met,^11^ and since the point estimate in this study is very close to the null, the conclusions cannot be materially altered if our statistical assumptions are not fully met.

## References

(1) Jamrozik K, Norman PE, Spencer CA, Parsons RW, Tuohy R, Lawrence-Brown MM et al. Screening for abdominal aortic aneurysm: lessons from a population-based study. Med J Aust 2000; 173:345-350.

(2) Miller SA, Dykes DD, Polesky HF. A simple salting out procedure for extracting DNA from human nucleated cells. Nucleic Acids Res 1988; 16:1215.

(3) Davey Smith G, Lawlor DA, Harbord R, Rumley A, Lowe GDO, Day INM et al. Association of C-reactive protein with blood pressure and hypertension: lifecourse confounding and Mendelian randomisation tests of causality. Arteriosclerosis Thrombosis & Vascular Biology 2005; 25:1051-1056.

(4) Lowe GD, Sweetnam PM, Yarnell JW, Rumley A, Rumley C, Bainton D et al. C-reactive protein, fibrin D-dimer, and risk of ischemic heart disease: the Caerphilly and Speedwell studies. Arterioscler Thromb Vasc Biol 2004; 24:1957-1962.

(5) Gimeno D, Brunner EJ, Lowe GDO, Rumley A, Marmot ME, Ferrie JE. Adult socioeconomic position, C-reactive protein and interleukin-6 in the Whitehall II prospective study. European Journal of Epidemiology 2007; In press.

(6) Casas JP, Shah T, Cooper J, Hawe E, McMahon AD, Gaffney D et al. Insights into the nature of the CRP-coronary event association using Medelian randomization. Int J Epidemiol 2006; 35:922-934.

(7) Lawlor DA, Harbord RM, Sterne JAC, Timpson NJ, Davey Smith G. Mendelian randomization: using genes as instruments for making causal inferences in epidemiology. Statistic in Medicine 2007; doi 10.1002/sim.3034.

(8) Thomas DC, Lawlor DA, Thompson JR. Re: Estimation of Bias in Nongenetic Observational Studies Using ''Mendelian Triangulation'' by Bautista et al**.** Annals of Epidemiology 2007;17:511-513.

(9) Fieller EC. Some problems in interval estimation. Journal of the Royal Statistical Scociety, Series B 1954; 16:175-185.

(10) Minelli C, Thompson JR, Tobin MD, Abrams KR. An integrated approach to the meta-analysis of genetic association studies using Mendelian randomization. Am J Epidemiol 2004; 160:445-452.

(11) Didelez V, Sheehan N. Mendelian randomization as an instrumental variable approach to causal inference. Statistical Methods in Medical Research 2007; 16:309-330.
